# Computed Tomography Measurement of Rib Cage Morphometry in Emphysema

**DOI:** 10.1371/journal.pone.0068546

**Published:** 2013-07-31

**Authors:** Nicola Sverzellati, Davide Colombi, Giorgia Randi, Antonio Pavarani, Mario Silva, Simon L. Walsh, Massimo Pistolesi, Veronica Alfieri, Alfredo Chetta, Mauro Vaccarezza, Marco Vitale, Ugo Pastorino

**Affiliations:** 1 Section of Diagnostic Imaging, Department of Surgery, University of Parma, Parma, Italy; 2 Department of Epidemiology, Mario Negri Institute, Milan, Italy; 3 Department of Radiology, St George's Hospital, London, United Kingdom; 4 Section of Respiratory Medicine, Department of Internal Medicine, University of Florence, Florence, Italy; 5 Department of Clinical and Experimental Medicine, Respiratory Disease and Lung Function Unit, University of Parma, Parma, Italy; 6 Department of Human, Social and Health Sciences, University of Cassino, Cassino, Italy; 7 Department of Biomedical, Biotechnological and Translational Sciences, Human Anatomy Section (S.Bi.Bi.T.), University of Parma, Parma, Italy; 8 Department Surgery, Section of Thoracic Surgery, Fondazione IRCCS Istituto Nazionale dei Tumori, Milan, Italy; Mayo Clinic College of Medicine, United States of America

## Abstract

**Background:**

Factors determining the shape of the human rib cage are not completely understood. We aimed to quantify the contribution of anthropometric and COPD-related changes to rib cage variability in adult cigarette smokers.

**Methods:**

Rib cage diameters and areas (calculated from the inner surface of the rib cage) in 816 smokers with or without COPD, were evaluated at three anatomical levels using computed tomography (CT). CTs were analyzed with software, which allows quantification of total emphysema (emphysema%). The relationship between rib cage measurements and anthropometric factors, lung function indices, and %emphysema were tested using linear regression models.

**Results:**

A model that included gender, age, BMI, emphysema%, forced expiratory volume in one second (FEV_1_)%, and forced vital capacity (FVC)% fit best with the rib cage measurements (R^2^ = 64% for the rib cage area variation at the lower anatomical level). Gender had the biggest impact on rib cage diameter and area (105.3 cm^2^; 95% CI: 111.7 to 98.8 for male lower area). Emphysema% was responsible for an increase in size of upper and middle CT areas (up to 5.4 cm^2^; 95% CI: 3.0 to 7.8 for an emphysema increase of 5%). Lower rib cage areas decreased as FVC% decreased (5.1 cm^2^; 95% CI: 2.5 to 7.6 for 10 percentage points of FVC variation).

**Conclusions:**

This study demonstrates that simple CT measurements can predict rib cage morphometric variability and also highlight relationships between rib cage morphometry and emphysema.

## Introduction

Anthropometric variables such as height, weight, sex, and age affect rib cage dimensions and morphology [1], [2], [3], [4]. However there is limited data on the range of rib cage morphometric variability as previous investigations have been performed in small study populations [1], [2], [3], [4], [5], [6]. Obtaining geometric data on the rib cage may be useful for biomechanical and surgical applications and may also increase the understanding of the relationships between rib cage morphometry, anthropometric parameters and thoracic abnormalities [6], [7], [8], [9].

Besides anthropometric factors, several lung diseases are independent determinants of the rib cage variability [5], [10], [11], [12], [13], [14]. Notably, chronic obstructive pulmonary disease (COPD) is a multi-compartmental disease which may cause hyperinflation of the lungs and musculoskeletal abnormalities [10], [11], [15], [16], [17]. As a consequence, COPD is likely to produce complex changes in the rib cage dimensions and shape [5], [15], [18], [19]. This has been suggested by previous investigations based on clinical assessment and chest radiographic techniques [20], [21].

However, the clinical assessment cannot quantify rib cage functional abnormities and measurements using conventional radiographic techniques are relatively inaccurate and hampered by the superimposition of bone and soft tissues. Recent technical developments allow a 3D reconstruction of the ribs from lateral and frontal chest radiographs, but these tools are not widely available [6], [22]. In contrast, CT may provide simple and precise measurements, which are capable of capturing rib cage changes due to several factors such as COPD.

The objective of this study was to assess the impact of anthropometric factors and COPD on the rib-cage variability using CT measurements in a large cohort of smokers.

## Methods

The study population consisted of 816 Caucasian patients (age range 39–86 years, mean 58.4±6.9 years, 559 men, 257 women). This cohort comprised two separate groups: 1) 725/816 (88.8%) subjects (485 men, 240 women, age range 49–75 years, mean 57.3 years±5.7) consecutively recruited by a lung cancer screening trial (MILD trial), between September 2005 and May 2006; 2) and 91/816 (9.9%) COPD patients (74 men, 17 women, age range 39–86 years, mean 67.4 years±8.2), who had undergone clinico-functional evaluation, but were not part of the lung cancer screening trial.

Eligibility criteria for the MILD trial included: men and women aged 49 to 75 years who had at least 20 pack-years of cigarette smoking and who currently smoked or smoked within the previous 10 years, with no history of cancer within the previous 5 years. Further details of MILD eligibility criteria have been previously described [23]. The original IRB approval and informed consent allowed use of MILD data for future research. Informed consent was obtained from all MILD participants for their information to be stored in the MILD database and used for research.

COPD patients were prospectively recruited in a tertiary care centre to participate in a study assessing the COPD-related emphysema phenotypes. This study was approved by the University Hospital of Parma's IRB which allows retrospective use of data for research purposes; informed consent was obtained from all patients for their information to be stored in the hospital database and used for research. All patients met GOLD criteria [compatible history and symptoms along with post-bronchodilator forced expiratory volume in one second (FEV_1_)/forced vital capacity (FVC) ≤0.7] [24].

### Pulmonary function testing

FEV_1_ and FVC were measured according to the American Thoracic Society (ATS) and European Respiratory Society (ERS) guidelines in MILD participants [25]. MILD participants with a pre-bronchodilator FEV_1_/FVC less than 0.7 (171/725, 23.5%) were defined as having modified COPD (mCOPD). MILD subjects with mCOPD were classified according to the GOLD stages (which ranks disease severity on a scale from 1–4, stage 4 being the most severe stage) as follows: 97 (56.7%) subjects with GOLD stage 1, 61 (35.7%) with GOLD stage 2, 12 (7%) with GOLD 3, 1 (0.6%) with GOLD stage 4 mCOPD. The 91 patients with COPD after post-bronchodilator spirometry were classified according to the GOLD stages as follows: 4 (4.4%) with GOLD stage 1, 47 (51.6%) with GOLD stage 2, 32 (35.2%) with GOLD stage 3, 8 (8.8%) with GOLD stage 4

### CT protocol

MILD participants were evaluated by using a 16-detector row CT scanner (Somatom Sensation 16, Siemens Medical Solutions, Forchheim, Germany), whereas COPD patients were studied with a 64-detector row CT scanner (64, Siemens Medical Solutions, Forchheim, Germany). All CT scans of the whole lung were acquired during one deep inspiratory breath-hold without the use of the intravenous contrast medium. Both scanners were calibrated daily using air to ensure measurements were accurate and consistent for all examinations. Standard CT parameters were used for both scanners: kV 120, effective mAs 30, individual detector collimation 0.75 mm, gantry rotation time 0.5 s, pitch 1.5.

### Rib cage analysis

The assessment of the rib cage morphometry was performed on CT reconstructions as follows: 1-mm-thick sections with a reconstruction increment of 1 mm and a soft kernel (B30f).

**Figure 1:**
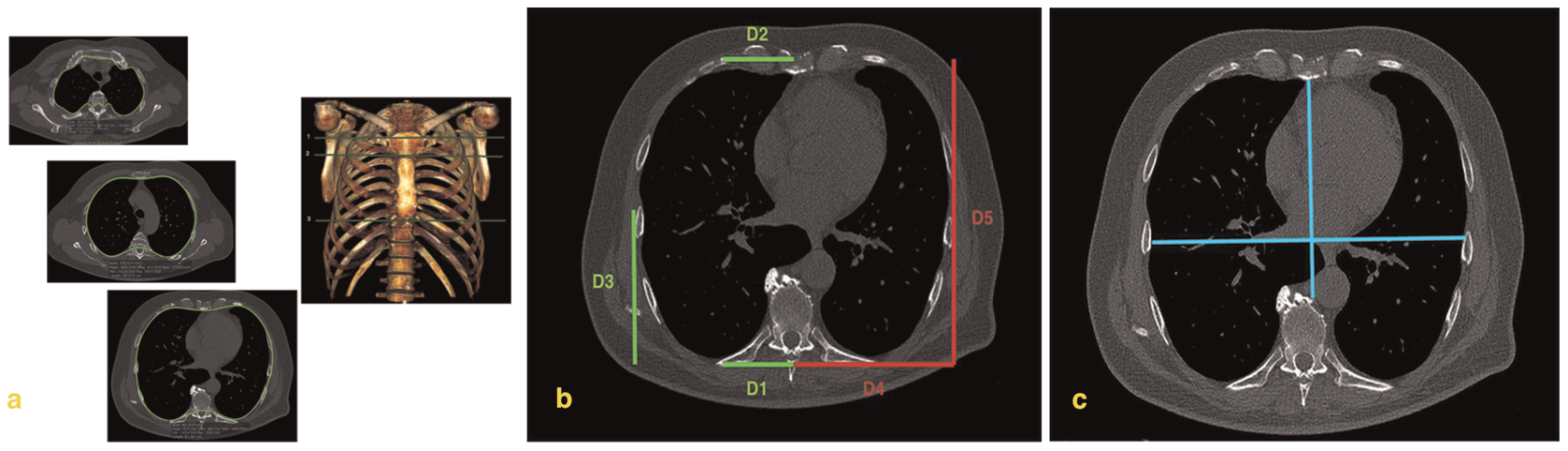
Examples illustrating the rib cage measurements. (**A**) Rib cage measurements were taken at three anatomical levels as follows: 1) sternal articulation of the first rib (upper level), 2) manubrio-sternal joint (middle level), and 3) xiphisternal joint (lower level). The areas burdens were manually traced by using a freehand electronic caliper (green line) along the inner surface of the rib cage. (**B**) For each hemithorax, several rib cage diameters (D) were measured by using the electronic caliper. To better display them, the diameters are shown separately. On the left side: D1 (from the most dorsal point of the rib and the apex of the vertebral spinous process); D2 (from the most ventral point of the rib cage to the sternal midpoint); D3 (from the most lateral point of the rib cage to the most lateral point of D4). On the right side: D4 (from the apex of vertebral spinous process to the lowest point of D3); D5 (from the most ventral point to the most dorsal point of the rib cage) diameters. (**C**) Haller's index was obtained by assessing the ratio between the mid-antero-posterior diameter (from the inner surface of the sternum to ventral surface of the vertebral body) and the global transverse diameter (from the most external mid-point of the rib of one side to the opposite one).

The first consecutive 100 CTs of the MILD cohort were transferred into two identical personal computers and reviewed, independently, by two operators (AP and DC, two residents with 2 years of experience in chest imaging) in order to evaluate the inter-operator variation related to rib cage morphometry assessment. The rib cage morphometry was assessed by using a Dicom viewer software validated for clinical purpose (OsiriX, 3.5.1 Imaging Processing Software 64 bit format). For each CT, internal rib cage measurements were taken at three anatomical levels as follows: 1) 1st sternocostal joint (upper level), 2) manubrio-sternal joint (middle level), and 3) xiphisternal joint (lower level) (Fig. 1A). These three anatomical levels were selected as they were shown to display the greatest inter-individual variability [26], [27].

Rib cage diameters, which best describe rib cage morphometry, were selected by an anatomist (MV, with 10 years of experience in research on rib cage morphometry) and a chest radiologist (NS) with expertise in anatomic and radiologic rib cage morphometry. Electronic calliper measurements were taken for each of these diameters within each hemithorax as follows (Fig. 1): dorsal transverse diameter (D1 - from the most dorsal point of the rib and the apex of the vertebral spinous process); ventral transverse diameter (D2 - from the most ventral point of the rib cage to the sternal midpoint); lower antero-posterior diameter (D3 - from the most lateral point of the rib cage to the most lateral point of D4); maximal transverse (D4 - from the apex of vertebral spinous process to the lowest point of D3) maximal antero-posterior (D5 - from the most ventral point to the most dorsal point of the rib cage) diameters (Fig. 1B). The Haller's index [28] - the ratio between the maximum transverse diameter of the chest (between the inner rib margins) and the minimum antero-posterior diameter (from the anterior aspect of the spine to the inner surface of the sternum) – was taken at the lower anatomical level to evaluate for the presence of pectus excavatum (Fig. 1C). A Haller's index greater than 3 was considered consistent with pectus excavatum. In addition, the total area included within the rib cage was also measured for each CT section. The areas burdens were manually traced by using the freehand electronic caliper along the inner surface of the rib cage (Fig. 1A).

After evaluating inter-operator variability for the measurements of the rib cage diameters and areas (see Results section and Table S1), the remaining study cases (n = 716) were independently reviewed by A.P. (350 CTs) and D.C. (366 CTs).

The operators also reviewed the CTs for the presence of interstitial lung disease or skeletal abnormality (e.g. extremely severe scoliosis) that could potentially influence the rib cage morphometry. No specific criteria were provided to the operators for these interpretations.

### Emphysema assessment

CT imaging data was reconstructed for the detection of pulmonary nodules (1-mm-thick sections with a reconstruction increment of 1 mm and a sharp kernel [medium-sharp kernel - B50f]) and transferred to a personal computer running MevisPULMO software (version 1.4, Fraunhofer MEVIS, Bremen, Germany). A quantitative analysis of emphysema was performed by one operator (NS). A 3×3 kernel-based axial Gauss smoothing algorithm was applied to minimise the noise in sharp kernel images. For the whole lung, emphysema extent was defined as the percentage of lung voxels less than or equal to -950 Hounsfield units (HU) [29].

### Statistical analysis

Inter-operator variability for the rib cage measurements were evaluated by the Bland and Altman method [30].

Differences between left and right hemithoraces measurements were assessed by the Student's paired t-test. The baseline characteristics of the study population were compared according to the presence or absence of COPD (i.e. patients with COPD vs. MILD subjects with mCOPD vs. MILD subjects without COPD) by using Chi-square test and analysis of variance (ANOVA) as appropriate.

Univariate linear regression analysis was used to examine the relationship between anthropometric factors, smoking history, functional data and emphysema %, and both rib cage areas and averaged (left and right) diameters. Multivariate linear regression was performed, entering univariate variables in a stepwise manner at the 0.05 significance level and removing them at the 0.10 level, in order to select factors most strongly associated with rib cage measurements. The selected factors were age, gender, body mass index (BMI), emphysema %, FEV_1_% and FVC%. We considered the goodness of fit of linear models by estimating the coefficient of determination (R^2^). Derived prediction equations were used to generate normal predicted values based on individual characteristics.

A p value less than 0.05 was considered statistically significant. All statistical analyses were performed using SAS Release 9.1 (SAS Institute Inc., Cary, North Carolina).

## Results

The inter-operator mean differences ranged from 0.03 mm to −4.9 mm and from −0.7 cm^2^ to −1.1 cm^2^ for the diameters and the areas respectively over the first 100 consecutive MILD CTs. Detailed inter-operator mean differences and levels of inter-observer variation for each rib cage measurement are summarized in the Table S1.

The baseline characteristics of the study population, stratified for the presence or absence of COPD are given in Table 1. There were three cases (0.4%) with interstitial lung disease consistent with usual interstitial pneumonia (UIP), although this was limited in extent. There were no major spinal abnormalities. For each anatomical level, both area and most diameters were different among the three subgroups (Table 1). The rib cage areas of subjects with COPD or mCOPD were significantly higher than those of normal subjects (p<.0001 to 0.03). No Haller's index greater than 3 was recorded.

**Table 1:** Baseline study population characteristics.

	PATIENTS WITH COPD (n = 91)	MILD SUBJECTS WITH MCOPD (n = 171)	NORMAL MILD SUBJECTS (n = 554)	p-value*	p-value (COPD vs. mCOPD)	p-value (COPD vs. normal)	p-value (mCOPD vs. normal)
Gender							
Male	74 (81.3%)	126 (73.7%)	359 (64.8%)				
Female	17 (18.7%)	45 (26.3%)	195 (35.2%)	0.0019	0.0019	0.166	0.031
Age	67.37 (8.20)	59.80 (6.55)	56.48 (5.27)	<.0001	<.0001	<.0001	<.0001
BMI	26.00 (4.14)	25.39 (3.93)	26.09 (6.23)	0.369	0.854	0.256	0.082
Pack-years	45.34 (25.10)	48.53 (25.12)	41.90 (19.15)	0.001	0.373	0.449	0.002
Smoking status							
Current	33 (53.2%)	125 (76.2%)	373 (69.9%)				
Former	29 (46.8%)	39 (23.8%)	161 (30.1%)	0.0035	0.008	0.001	0.115
FEV_1_%	52.91 (16.75)	81.23 (20.29)	102.28 (14.71)	<.0001	<.0001	<.0001	<.0001
FVC%	75.03 (18.42)	103.82 (22.78)	105.96 (16.84)	<.0001	<.0001	<.0001	0.266
FEV_1_/FVC %	54.75 (10.13)	61.57 (8.05)	79.57 (11.01)	<.0001	<.0001	<.0001	<.0001
Emphysema %	14.2 (10.6)	7.6 (5.3)	4.2 (3.7)	<.0001	<.0001	<.0001	<.0001
AREAS							
U	199.09 (29.06)	184.56 (31.89)	176.76 (32.61)	<.0001	<.0001	<.0001	0.006
M	306.63 (35.65)	311.32 (40.60)	296.93 (40.12)	<.0001	0.031	0.354	<.0001
L	458.25 (58.33)	446.53 (66.35)	434.78 (66.33)	0.002	0.002	0.157	0.043
DIAMETERS							
D1_U	51.65 (5.19)	53.19 (5.20)	52.64 (5.30)	0.080	0.099	0.023	0.233
D1_M	55.26 (5.14)	57.41 (5.43)	56.52 (5.20)	0.006	0.032	0.002	0.053
D1_L	62.71 (5.99)	64.22 (6.41)	63.36 (6.16)	0.134	0.348	0.065	0.116
D2_U	53.70 (6.71)	57.33 (8.45)	56.49 (8.88)	0.004	0.001	0.000	0.276
D2_M	41.65 (8.11)	45.39 (8.59)	46.33 (8.44)	<.0001	<.0001	0.001	0.203
D2_L	53.60 (11.50)	61.36 (10.92)	61.09 (10.73)	<.0001	<.0001	<.0001	0.776
D3_U	54.85 (8.46)	53.95 (8.72)	52.52 (8.81)	0.022	0.019	0.425	0.063
D3_M	75.85 (9.70)	78.82 (9.99)	76.17 (10.08)	0.008	0.777	0.021	0.003
D3_L	103.74 (13.90)	103.08 (11.79)	101.98 (12.67)	0.346	0.228	0.690	0.312
D4_U	98.86 (8.70)	100.68 (9.52)	99.13 (9.37)	0.136	0.800	0.130	0.059
D4_M	114.12 (7.75)	118.64 (8.26)	116.68 (8.04)	<.0001	0.005	<.0001	0.006
D4_L	131.04 (9.30)	134.75 (10.50)	133.62 (10.07)	0.018	0.023	0.005	0.201
D5_U	120.87 (12.34)	111.27 (12.92)	108.27 (12.57)	<.0001	<.0001	<.0001	0.007
D5_M	163.70 (13.39)	162.41 (13.28)	157.27 (13.50)	<.0001	<.0001	0.456	<.0001
D5_L	207.81 (19.10)	200.17 (18.01)	195.73 (19.00)	<.0001	<.0001	0.002	0.007
Haller's Index	1.73 (0.27)	1.92 (0.28)	1.96 (0.27)	<.0001	<.0001	<.0001	0.092

Notes: D = diameter; U = upper; M = middle; L = lower;

* p-value indicates the probability of the null hypothesis when comparing patients with COPD, MILD subjects with modified COPD (mCOPD), and normal MILD subjects by using one-way ANOVA analysis.

Differences between right and left diameters ranged from 0.7 mm to 4 mm (p<0. 0001 to 0.017; Table 2). The greatest asymmetry was recorded for D2 and D5, which were larger on the right side at the lower and the upper anatomical level, respectively.

**Table 2:** Differences between left and right side of each measures in all subjects, with the p-value of the difference.

DIAMETERS	Section	Mean	SD	p-value*
D1	U	1.10	5.0	<.0001
	M	1.13	4.3	<.0001
	L	2.12	4.6	<.0001
D2	U	−0.02	12.7	0.967
	M	0.00	13.4	0.998
	L	−4.01	16.0	<.0001
D3	U	−0.83	7.6	<.0001
	M	−0.16	8.6	0.587
	L	−1.72	13.7	<.0001
D4	U	−1.55	7.2	<.0001
	M	−0.38	7.3	0.139
	L	−0.75	8.9	0.017
D5	U	−2.65	9.7	<.0001
	M	−1.29	5.4	<.0001
	L	0.67	6.3	0.003

Notes: SD = standard deviation;

* p-value was estimated by paired t-test.

A regression model which included gender, age, and, emphysema%, FEV_1_%, and FVC% fit best with the rib cage measurements. Table 3 shows the partial regression coefficients (β) for all the included model variables. Overall, the coefficients were greater for the rib cage measurements at the lower anatomical level, explaining up to 64% of rib cage variation.

**Table 3:** Partial regression coefficient (β) and 95% confidence intervals (95%CI) of area variation at different lung sections by selected variables.

		U		M		L	
		β	95%CI	β	95%CI	β	95%CI
Areas	Gender§	**26.0**	**(30.8,21.2)**	**45.0**	**(50.5,39.6)**	**105.3**	**(111.7,98.8)**
	Age	0.4	(0.0,0.7)	0.3	(−0.1,0.7)	0.5	(0.0,0.9)
	BMI	−0.4	(−0.7,0.0)	0.2	(−0.2,0.7)	**2.8**	**(2.3,3.3)**
	Emphysema%*	**3.9**	**(1.8,6.0)**	**5.4**	**(3.0,7.8)**	2.4	(−0.4,5.3)
	FEV_1_%**	−1.5	(−3.2,0.3)	−0.2	(−2.2,1.8)	−0.4	(−2.7,2.0)
	FVC%**	1.3	(−0.6,3.2)	**2.8**	**(0.7,4.9)**	**5.1**	**(2.5,7.6)**
	R^2^		20.6%		31.7%		64.0%
D1	Gender§	**3.90**	**(4.71,3.09)**	**4.90**	**(5.68,4.12)**	**7.21**	**(8.06,6.35)**
	Age	**−0.08**	**(−0.14,−0.02)**	**−0.07**	**(−0.13,−0.01)**	−0.01	(−0.08,0.05)
	BMI	−0.06	(−0.12,0.01)	0.00	(−0.06,0.06)	0.00	(−0.07,0.07)
	Emphysema%*	0.24	(−0.12,0.59)	0.13	(−0.21,0.47)	0.13	(−0.25,0.50)
	FEV_1_%**	−0.01	(−0.31,0.29)	0.04	(−0.25,0.32)	0.13	(−0.18,0.45)
	FVC%**	0.13	(−0.18,0.45)	0.15	(−0.15,0.45)	0.27	(−0.06,0.61)
	R^2^		12.0%		18.5%		28.3%
D2	Gender§	**8.12**	**(9.34,6.89)**	**3.98**	**(5.32,2.63)**	**8.87**	**(10.53,7.22)**
	Age	−0.07	(−0.16,0.03)	**−0.11**	**(−0.21,−0.01)**	−0.04	(−0.17,0.08)
	BMI	**0.17**	**(0.07,0.27)**	**0.17**	**(0.06,0.28)**	0.12	(−0.02,0.25)
	Emphysema%*	−0.37	(−0.91,0.17)	−0.58	(−1.17,0.01)	−0.69	(−1.42,0.04)
	FEV_1_%**	0.09	(−0.35,0.54)	0.13	(−0.37,0.62)	0.61	(0.00,1.21)
	FVC%**	0.15	(−0.33,0.63)	0.09	(−0.44,0.61)	0.41	(−0.24,1.06)
	R^2^		22.0%		8.3%		16.7%
D3	Gender§	**3.68**	**(5.08,2.28)**	**5.05**	**(6.64,3.46)**	**11.83**	**(13.63,10.04)**
	Age	0.04	(−0.07,0.14)	−0.01	(−0.13,0.11)	0.05	(−0.08,0.19)
	BMI	−0.10	(−0.21,0.02)	−0.02	(−0.15,0.11)	**0.36**	**(0.22,0.51)**
	Emphysema%*	**0.75**	**(0.14,1.37)**	1.08	(0.38,1.78)	0.61	(−0.18,1.40)
	FEV_1_%**	−0.12	(−0.63,0.39)	0.16	(−0.43,0.74)	0.30	(−0.36,0.96)
	FVC%**	0.05	(−0.49,0.60)	0.12	(−0.50,0.74)	0.35	(−0.35,1.05)
	R^2^		6.6%		7.4%		23.9%
D4	Gender§	**6.30**	**(7.74,4.86)**	**7.77**	**(8.94,6.60)**	**14.50**	**(15.70,13.30)**
	Age	−0.10	(−0.20,0.01)	**−0.12**	**(−0.21,−0.03)**	−0.05	(−0.14,0.03)
	BMI	**−0.15**	**(−0.26,−0.03)**	−0.03	(−0.13,0.06)	**0.29**	**(0.20,0.39)**
	Emphysema%*	**1.02**	**(0.39,1.65)**	**1.01**	**(0.49,1.52)**	0.19	(−0.34,0.71)
	FEV_1_%**	−0.07	(−0.59,0.46)	0.23	(−0.20,0.66)	0.34	(−0.11,0.78)
	FVC%**	0.33	(−0.23,0.89)	0.39	(−0.07,0.85)	**0.77**	**(0.30,1.24)**
	R^2^		12.0%		22.3%		47.7%
D5	Gender§	**7.55**	**(9.51,5.58)**	**12.27**	**(14.23,−10.31)**	**26.08**	**(28.26,23.89)**
	Age	**0.28**	**(0.13,0.43)**	**0.30**	**(0.16,0.45)**	**0.29**	**(0.13,0.45)**
	BMI	−0.11	(−0.27,0.05)	0.10	(−0.06,0.25)	**0.75**	**(0.57,0.93)**
	Emphysema%*	**1.44**	**(0.58,2.29)**	1.55	(0.69,2.40)	0.87	(−0.08,1.83)
	FEV_1_%**	**−0.76**	**(−1.48,−0.04)**	−0.23	(−0.94,0.49)	−0.55	(−1.35,0.25)
	FVC%**	0.52	(−0.25,1.28)	**0.93**	**(0.17,1.70)**	**1.29**	**(0.44,2.14)**
	R^2^		17.5%		24.5%		51.2%

Notes: numbers in bold characters are statistically significant;

§ male as compared with female;

* 5 percentage points variation;

** 10 percentage points variation.

Gender was the strongest predictor of both diameter and area at all CT levels - rib cage measurements were greater for men with the most striking gender difference (105.3 cm^2^, 95% CI: 111.7 to 98.8) reported for the lower rib cage area. Gender was the only independent predictor of the Haller's index, although this association was weak (0.11, 95% CI: 0.07 to 0.15 for women as compared to men). Although age and BMI had a similar impact on rib cage measurements at the upper and middle levels, the impact of BMI was greater for the lower area measurement which increased as BMI increased (Table 3).

**Figure 2:**
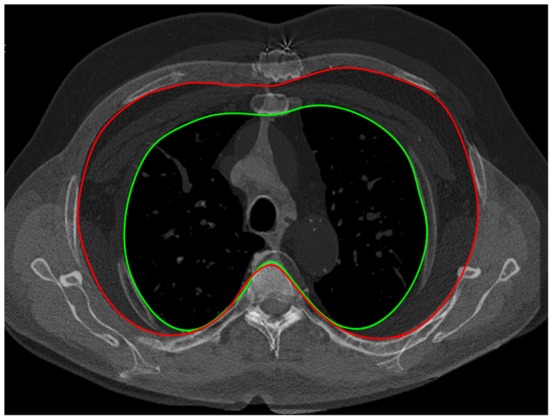
Example showing the independent effect of emphysema. Two overlapping upper (second level) CT slices of two different male subjects with similar demographic characteristics: the subject with the smaller area (green line) had an emphysema extent of 2.1%, whereas the one with the larger area (red line) had an emphysema extent of 35%.

Emphysema% influenced diameters and areas more than FEV_1_% and FVC% at the upper anatomical level (Table 3). Specifically, for an emphysema increase of 5%, upper and middle areas respectively increased by 3.9 cm^2^ (95% CI: 1.8 to 6.0) and 5.4 cm2 (95% CI: 3 to 7.8) respectively. The effect of emphysema% did not substantially change when adjusted only for age, gender and BMI. Rib cage changes according to the proportional increase of emphysema% are displayed in Fig. 2.

The selected model showed a proportional relationship between FVC% and both middle and lower areas (Table 3). Specifically, for a 10% decrease in FVC%, the middle area decreased by 2.8 cm^2^ (95% CI: 0.7 to 4.9) and lower area by 5.1 cm^2^ (95% CI: 2.5 to 7.6).

**Figure 3:**
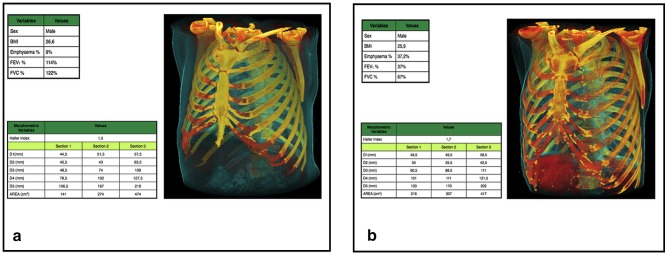
Rib cage morphometric differences between a normal 58-year-old male smoker (A) and a 61-year-old male COPD patient with severe emphysema (B). Both detailed measurements and the 3D reformation display the main morphologic differences: larger upper rib cage dimensions and smaller lower rib cage dimensions in COPD. Overall, the morphometric changes in COPD contribute to the “barrel chest” configuration.

FEV_1_% was not significantly related with most measurements (p>0.05), and this was true even for values lower than 50% predicted (as found by a post-hoc subanalysis). However, by eliminating the potential confounding interaction with emphysema extent and FVC% (i.e. by adjusting only for gender, age and BMI), the effect of decreasing FEV_1_% on the rib cage lower area was similar to that observed for FVC%: for a 10% decrease of FEV_1_%, the lower area decreased of 2.8 cm^2^ (95% CI: 1.5 to 4.2). The relationship between rib cage variation and functional decline is exemplified in Fig. 3.

Predicted rib cage measurements based on individual characteristics as derived by the model's prediction equations can be calculated using the online interactive File S1.

## Discussion

To the best of our knowledge, this is the first normative study which provides ranges of rib cage morphometry in a large series of adult smokers as well as novel data about rib cage variation due to anthropometric and lung disease-related factors.

In line with prior investigations assessing rib cage diameters on the chest radiograph, we found that gender had the strongest impact on rib cage dimension [3], [4]. Bellemare et al. reported that cross-section areas as well as anterior–posterior and transverse diameters were significantly smaller in females than in males with the same anthropometric characteristics as assessed at anatomical levels similar to those of the current study. Such a difference is explained by a greater inclination of the ribs in females [4].

At upper and middle levels, the impact of emphysema% on rib cage morphometry was greater than that of age and BMI. To our knowledge this is the first study evaluating the relationship between emphysema and rib cage morphometric variability. This was made possible by CT, which is considered the gold standard for quantifying extent of emphysema. The effect of increasing emphysema % on rib cage morphometry was striking - rib cage area as well as both antero-posterior and maximal transverse diameters at the upper-middle levels increased as emphysema % increased. We speculate that the bony rib cage undergoes chronic adaptation to more severe emphysema in the upper lobes.

The relationship between rib cage variation and COPD is controversial [5], [10], [11], [19], [31], [32], [33]. It was traditionally believed that patients with hyperinflation and COPD accommodate part of the increased lung volume by expanding the rib cage [5], [34]. By matching seven normal with seven COPD subjects, Cassart et al. showed an increase in antero-posterior but not in trasverse diameters on CT such that the rib cage adopted a more circular shape among subjects with COPD [5]. By contrast, other studies measuring several diameters on chest radiography found that when compared to sex-, age-, and height-matched normal subjects, the rib cage of COPD patients remained normal in size and shape, with the exception of an increase of cranio-caudal diameter due to a caudal displacement of the diaphragm [3], [35], [36]. We found that when adjusting for anthropometric variables and emphysema%, decreasing FEV_1_% did not produce any relevant change for rib cage areas. This suggests that in COPD, the parenchymal destruction associated with emphysema may have a greater impact on rib cage morphometric variability than conductive airway abnormalities. This is in keeping with the early observation that, for a given level of airflow obstruction, patients with a emphysema dominant subtype are more likely demonstrate a ‘barrel chest’ rib cage configuration, than those with chronic bronchitis and/or small airways disease [37].

Nevertheless, we found significant relationships between FVC% and FEV_1_% decrease (the latter when adjusted only for anthropometric variables) and middle-lower rib cage areas and lateral diameters reduction. It is well known that patients with reduced lung function can display a reduced range of motion of the diaphragm, thus impeding the ability of the rib cage to enlarge during full inspiration. This observation is consistent with the so called Hoover's sign, so-called because of the inward motion of the lateral diameter of the lower rib cage during the inspiratory phase [38], [39]. This has been attributed to direct diaphragmatic traction on the lower rib cage margin, when the diaphragm is flattened in conditions associated with hyperinflation [40], [41]. COPD may disadvantage the respiratory muscles by impairing their capacity to generate force [17], [42].

Our study has several limitations. Although large, our study population size is still insufficient to fully explore ranges of rib cage morphometry which are highly variable. Rib cage measurements were obtained from CT examinations in a supine decubitus position during deep inspiratory. Such conditions influence the rib cage morphometry limiting transpositions or matching with measurements directly taken from the body or the upright chest radiography. CT data acquisitions were not spirometrically controlled, which would have enabled a standardization of the inspiratory level. The assessment of the functional associations may be biased as for the majority of subjects functional reversibility data were not available; however, the possibility that these subjects were misclassified is minimal mainly based on their heavy smoking history. Diameters and areas were measured only at three pre-selected anatomical levels, but more sections are needed to get more complete information of the entire rib cage. Rib cage diameters might also be influenced by skeletal disease (e.g. scoliosis, osteoporosis etc.) which was not fully assessed in the present study. Nevertheless averaging left and right hemithoracic measurements, should have reduced bias associated with skeletal abnormalities. Furthermore, the mild degree of asymmetry reported between left and right side suggests that severe scoliosis was not frequent in our study population. The relatively low prevalence of more severe GOLD stages limits the interpretation of the relationship between airflow limitation and the rib cage morphometry.

In conclusion, this study provides ranges of several rib cage measurements in smokers with or without COPD. We have also revealed an important independent relationship with emphysema. Lastly, our statistical model can only partly explain the rib cage variation, suggesting that there are other factors, which are important and should be the focus of further studies.

## Supporting Information

Table S1 Mean Differences, P-Values, Standard Deviations and Associated Limits of Agreement (LOA) for the rib cage diameters and areas.
(DOCX)

File S1 Instructions: users can change values in the green column (above) and obtain a prediction of the rib cage measurements in the three blue columns (below); the other file sheets (i.e. areas, diameters and other measurements have not to be removed because they include corresponding prediction equations derived from the multivariate analysis).
(XLS)

## References

[1] Takahashi E, Atsumi H (1955) Age differences in thoracic form as indicated by thoracic index. Hum Biol 27: 65–74. 14381054

[2] Lennon EA, Simon G (1965) The height of the diaphragm in the chest radiograph of normal adults. Br J Radiol 38: 937–943. 5850454 10.1259/0007-1285-38-456-937

[3] Bellemare JF, Cordeau MP, Leblanc P, Bellemare F (2001) Thoracic dimensions at maximum lung inflation in normal subjects and in patients with obstructive and restrictive lung diseases. Chest 119: 376–386. 11171712 10.1378/chest.119.2.376

[4] Bellemare F, Jeanneret A, Couture J (2003) Sex differences in thoracic dimensions and configuration. Am J Respir Crit Care Med 168: 305–312. 12773331 10.1164/rccm.200208-876OC

[5] Cassart M, Gevenois PA, Estenne M (1996) Rib cage dimensions in hyperinflated patients with severe chronic obstructive pulmonary disease. Am J Respir Crit Care Med 154: 800–805. 8810622 10.1164/ajrccm.154.3.8810622

[6] Gayzik FS, Yu MM, Danelson KA, Slice DE, Stitzel JD (2008) Quantification of age-related shape change of the human rib cage through geometric morphometrics. J Biomech 41: 1545–1554. 18384793 10.1016/j.jbiomech.2008.02.006

[7] Girotti P, Leo F, Bravi F, Tavecchio L, Spano A, et al (2011) The “rib-like” technique for surgical treatment of sternal tumors: lessons learned from 101 consecutive cases. Ann Thorac Surg 92: 1208–1215 discussion 1215–1206. 21958766 10.1016/j.athoracsur.2011.05.016

[8] Leo F, Girotti P, Tavecchio L, Conti B, Delledonne V, et al (2010) Anterior diaphragmatic plication in mediastinal surgery: the “reefing the mainsail” technique. Ann Thorac Surg 90: 2065–2067. 21095375 10.1016/j.athoracsur.2010.02.043

[9] Lee M, Kelly DW, Steven GP (1995) A model of spine, ribcage and pelvic responses to a specific lumbar manipulative force in relaxed subjects. J Biomech 28: 1403–1408. 8522552 10.1016/0021-9290(95)00001-x

[10] Aliverti A (2008) Lung and chest wall mechanics during exercise: effects of expiratory flow limitation. Respir Physiol Neurobiol 163: 90–99. 18721912 10.1016/j.resp.2008.07.025

[11] Aliverti A, Stevenson N, Dellaca RL, Lo Mauro A, Pedotti A, et al (2004) Regional chest wall volumes during exercise in chronic obstructive pulmonary disease. Thorax 59: 210–216. 14985554 10.1136/thorax.2003.011494 PMC1746979

[12] Simon BA, Christensen GE, Low DA, Reinhardt JM (2005) Computed tomography studies of lung mechanics. Proc Am Thorac Soc 2: 517–521, 506–517. 16352757 10.1513/pats.200507-076DS PMC2713339

[13] Ben-Haim SA, Saidel GM (1989) Chest wall mechanics: effects of acute and chronic lung disease. J Biomech 22: 559–564. 2530231 10.1016/0021-9290(89)90007-9

[14] Fauroux B, Aubertin G, Cohen E, Clement A, Lofaso F (2009) Sniff nasal inspiratory pressure in children with muscular, chest wall or lung disease. Eur Respir J 33: 113–117. 18799509 10.1183/09031936.00050708

[15] Ley-Zaporozhan J, Ley S, Kauczor HU (2008) Morphological and functional imaging in COPD with CT and MRI: present and future. Eur Radiol 18: 510–521. 17899100 10.1007/s00330-007-0772-1

[16] Malaguti C, Rondelli RR, de Souza LM, Domingues M, Dal Corso S (2009) Reliability of chest wall mobility and its correlation with pulmonary function in patients with chronic obstructive pulmonary disease. Respir Care 54: 1703–1711. 19961637

[17] De Troyer A, Wilson TA (2009) Effect of acute inflation on the mechanics of the inspiratory muscles. J Appl Physiol 107: 315–323. 19265064 10.1152/japplphysiol.91472.2008

[18] De Troyer A (2012) Respiratory effect of the lower rib displacement produced by the diaphragm. J Appl Physiol 112: 529–534. 22134697 10.1152/japplphysiol.01067.2011

[19] Jubran A, Tobin MJ (1992) The effect of hyperinflation on rib cage-abdominal motion. Am Rev Respir Dis 146: 1378–1382. 1456551 10.1164/ajrccm/146.6.1378

[20] Marazzini L, Rizzato GF (1970) Relative contribution of rib cage and abdomen-diaphragm to the variation of lung volume in emphysema. Respiration 27: 105–119. 5438653 10.1159/000192676

[21] Sharp JT, Beard GA, Sunga M, Kim TW, Modh A, et al (1986) The rib cage in normal and emphysematous subjects: a roentgenographic approach. J Appl Physiol 61: 2050–2059. 3100493 10.1152/jappl.1986.61.6.2050

[22] Mitton D, Zhao K, Bertrand S, Zhao C, Laporte S, et al (2008) 3D reconstruction of the ribs from lateral and frontal X-rays in comparison to 3D CT-scan reconstruction. J Biomech 41: 706–710. 17981286 10.1016/j.jbiomech.2007.09.034

[23] Pastorino U, Rossi M, Rosato V, Marchiano A, Sverzellati N, et al (2012) Annual or biennial CT screening versus observation in heavy smokers: 5-year results of the MILD trial. Eur J Cancer Prev 21: 308–315. 22465911 10.1097/CEJ.0b013e328351e1b6

[24] ATS statement: guidelines for the six-minute walk test. Am J Respir Crit Care Med 166: 111–117. 12091180 10.1164/ajrccm.166.1.at1102

[25] Standards for the diagnosis and care of patients with chronic obstructive pulmonary disease. American Thoracic Society. Am J Respir Crit Care Med 152: S77–121. 7582322

[26] Testut L (1899) Traite D'Anatomy Humaine. Paris: Octave Doin.

[27] Standring S (2008) GRAY'S ANATOMY: THE ANATOMICAL BASIS OF CLINICAL PRACTICE: Churchill-Livingstone Elsevier.

[28] Haller JA Jr, Kramer SS, Lietman SA (1987) Use of CT scans in selection of patients for pectus excavatum surgery: a preliminary report. J Pediatr Surg 22: 904–906. 3681619 10.1016/s0022-3468(87)80585-7

[29] Gevenois PA, De Vuyst P, de Maertelaer V, Zanen J, Jacobovitz D, et al (1996) Comparison of computed density and microscopic morphometry in pulmonary emphysema. Am J Respir Crit Care Med 154: 187–192. 8680679 10.1164/ajrccm.154.1.8680679

[30] Bland JM, Altman DG (1986) Statistical methods for assessing agreement between two methods of clinical measurement. Lancet 1: 307–310. 2868172

[31] Decramer M (1997) Hyperinflation and respiratory muscle interaction. Eur Respir J 10: 934–941. 9150337

[32] Briscoe WA, Dubois AB (1958) The relationship between airway resistance, airway conductance and lung volume in subjects of different age and body size. J Clin Invest 37: 1279–1285. 13575526 10.1172/JCI103715 PMC1062796

[33] Binazzi B, Bianchi R, Romagnoli I, Lanini B, Stendardi L, et al (2008) Chest wall kinematics and Hoover's sign. Respir Physiol Neurobiol 160: 325–333. 18088571 10.1016/j.resp.2007.10.019

[34] Rothpearl A, Varma AO, Goodman K (1988) Radiographic measures of hyperinflation in clinical emphysema. Discrimination of patients from controls and relationship to physiologic and mechanical lung function. Chest 94: 907–913. 3180893 10.1378/chest.94.5.907

[35] Kilburn KH, Asmundsson T (1969) Anteroposterior chest diameter in emphysema. From maxim to measurement. Arch Intern Med 123: 379–382. 5778119

[36] Walsh JM, Webber CL Jr, Fahey PJ, Sharp JT (1992) Structural change of the thorax in chronic obstructive pulmonary disease. J Appl Physiol 72: 1270–1278. 1592714 10.1152/jappl.1992.72.4.1270

[37] Pierce JA, Ebert RV (1958) The barrel deformity of the chest, the senile lung and obstructive pulmonary emphysema. Am J Med 25: 13–22. 13559256 10.1016/0002-9343(58)90193-1

[38] Johnston CR 3rd, Krishnaswamy N, Krishnaswamy G (2008) The Hoover's Sign of Pulmonary Disease: Molecular Basis and Clinical Relevance. Clin Mol Allergy 6: 8. 18775073 10.1186/1476-7961-6-8 PMC2546439

[39] Bruyneel M, Jacob V, Sanida C, Ameye L, Sergysels R, et al (2011) Hoover's sign is a predictor of airflow obstruction severity and is not related to hyperinflation in chronic obstructive pulmonary disease. Eur J Intern Med 22: e115–118. 22075295 10.1016/j.ejim.2011.08.020

[40] Gilmartin JJ, Gibson GJ (1986) Mechanisms of paradoxical rib cage motion in patients with chronic obstructive pulmonary disease. Am Rev Respir Dis 134: 683–687. 2945502 10.1164/arrd.1986.134.4.683

[41] Leduc D, Cappello M, Gevenois PA, De Troyer A (2012) Mechanism of the lung-deflating action of the canine diaphragm at extreme lung inflation. J Appl Physiol 112: 1311–1316. 22323651 10.1152/japplphysiol.01422.2011

[42] Priori R, Aliverti A, Albuquerque AL, Quaranta M, Albert P, et al (2013) The effect of posture on asynchronous chest wall movement in COPD. J Appl Physiol 114: 1066–1075. 23412901 10.1152/japplphysiol.00414.2012

