# Supplementary material for: Computed Tomography Measurement of Rib Cage Morphometry in Emphysema
Source: PLoS One. 2013 Jul 31;8(7):e68546. doi: 10.1371/journal.pone.0068546 (PMC3729561; doi:10.1371/journal.pone.0068546)
Supplement: Table S1 — Mean Differences, P-Values, Standard Deviations and Associated Limits of Agreement (LOA) for the rib cage diameters and areas. (DOCX) [file pone.0068546.s001.docx]

**Table S1.**

| **Variable** | **Mean difference** | **p-value** | **SD** | **Lower LOA**  **(mean-2SD)** | **Upper LOA**  **(mean+2SD)** | **Range** |
| --- | --- | --- | --- | --- | --- | --- |
| **DIAMETERS** |  |  |  |  |  |  |
| D1rU | -1.4 | 0.0002 | 3.7 | -8.8 | 5.9 | 14.7 |
| D1rM | -0.8 | 0.0044 | 2.8 | -6.4 | 4.8 | 11.3 |
| D1rL | -1.2 | 0.0247 | 5.3 | -11.9 | 9.5 | 21.4 |
| D1lU | -0.5 | 0.0532 | 2.6 | -5.8 | 4.8 | 10.6 |
| D1lM | -1.7 | <.0001 | 2.4 | -6.6 | 3.2 | 9.8 |
| D1lL | -1.5 | <.0001 | 2.8 | -7.1 | 4.2 | 11.4 |
| D2rU | -1.7 | 0.0103 | 6.5 | -14.8 | 11.4 | 26.2 |
| D2rM | -4.9 | <.0001 | 6.7 | -18.4 | 8.6 | 26.9 |
| D2rL | -4.6 | <.0001 | 8.1 | -20.9 | 11.7 | 32.6 |
| D2lU | -1.3 | 0.0245 | 5.8 | -12.9 | 10.3 | 23.3 |
| D2lM | -4.4 | <.0001 | 10.1 | -24.6 | 15.8 | 40.5 |
| D2lL | 0.03 | 0.9539 | 5.2 | -10.3 | 10.4 | 20.7 |
| D3rU | -4.520 | <.0001 | 5.4 | -15.3 | 6.2 | 21.5 |
| D3rM | -3.5 | <.0001 | 3.9 | -11.4 | 4.4 | 15.8 |
| D3rL | -3.6 | <.0001 | 7.2 | -18 | 10.9 | 28.9 |
| D3lU | -2.6 | <.0001 | 4.3 | -11.3 | 6 | 17.3 |
| D3lM | -1.3 | 0.0093 | 4.8 | -10.8 | 8.3 | 19.1 |
| D3lL | -1.6 | 0.0128 | 6.3 | -14.3 | 11.1 | 25.4 |
| D4rU | -1.4 | <.0001 | 2.1 | -5.6 | 2.8 | 8.4 |
| D4rM | -1.2 | <.0001 | 1.6 | -4.4 | 1.9 | 6.4 |
| D4rL | -1.3 | <.0001 | 1.8 | -4.9 | 2.4 | 7.4 |
| D4lU | -1.2 | <.0001 | 2.2 | -5.6 | 3.3 | 8.9 |
| D4lM | -1.250 | <.0001 | 2.4 | -6.2 | 3.7 | 9.9 |
| D4lL | -0.9 | <.0001 | 1.8 | -4.7 | 2.8 | 7.6 |
| D5rU | -3.8 | <.0001 | 5.2 | -14.2 | 6.5 | 20.7 |
| D5rM | -1.3 | <.0001 | 2.9 | -7.2 | 4.6 | 11.8 |
| D5rL | -1.7 | <.0001 | 1.2 | -4.2 | 0.7 | 4.8 |
| D5lU | -3.4 | <.0001 | 4.7 | -12.8 | 6 | 18.8 |
| D5lM | -1.5 | <.0001 | 1.7 | -4.8 | 1.9 | 6.7150 |
| D5lL | -2.2 | <.0001 | 4.4 | -11 | 6.6 | 17.6 |
| Haller's index | -0.1 | 0.09 | 2.1 | -0.4 | 0.8 |  |
| **AREAS** |  |  |  |  |  |  |
| U | -0.700 | 0.1184 | 4.4 | -9.5876 | 8.2 | 17.8 |
| M | -1.180 | 0.0015 | 3.6 | -8.4240 | 6.1 | 14.5 |
| L | -0.570 | 0.1701 | 4.1 | -8.8199 | 7.7 | 16.5 |

Notes. D: diameter; l: left side; r: right; U: upper level; M: middle level; L: lower level; LOA: Limits of Agreement. Size units for diameters and areas were respectively millimetres (mm) and square centimetres (cm^2^).
